# P17 Influence of drug infusion pumps compared with an electronic prescribing and medicines administration (ePMA) system on vancomycin pharmacokinetic model fit: implications for bacterial resistance breakpoints in critically ill adults and children

**DOI:** 10.1093/jacamr/dlae217.021

**Published:** 2025-01-23

**Authors:** Robert Oakley, Divyen Vanniasegaram, Claire Ostrowska, Cristina Da Silva, Sophie Cookes, Atif Mian, Agnieszka Sprawka-Koszel, Harriet Davidson, Qurban Mohebi, Reya Shah, Soumendu Manna, Joseph Standing, Dagan O Lonsdale

**Affiliations:** St George’s, University of London, London, UK; St George’s University Hospitals NHS Foundation Trust, London, UK; St George’s, University of London, London, UK; St George’s University Hospitals NHS Foundation Trust, London, UK; St George’s University Hospitals NHS Foundation Trust, London, UK; St George’s University Hospitals NHS Foundation Trust, London, UK; St George’s University Hospitals NHS Foundation Trust, London, UK; St George’s University Hospitals NHS Foundation Trust, London, UK; St George’s University Hospitals NHS Foundation Trust, London, UK; St George’s, University of London, London, UK; St George’s University Hospitals NHS Foundation Trust, London, UK; St George’s University Hospitals NHS Foundation Trust, London, UK; St George’s, University of London, London, UK; St George’s University Hospitals NHS Foundation Trust, London, UK; St George’s University Hospitals NHS Foundation Trust, London, UK; University College London Great Ormond Street Institute of Child Health, London, UK; St George’s, University of London, London, UK; St George’s University Hospitals NHS Foundation Trust, London, UK

## Abstract

**Background:**

Pharmacokinetic models contribute to calculating resistance suppression breakpoints and optimizing antibiotic doses. Antibiotic administration details, blood concentrations and sample times, infection and patient characteristics are required. Inputted data is assumed errorless. Vancomycin is a glycopeptide antibiotic administered by intermittent or continuous infusion. The vancomycin pharmacokinetic/pharmacodynamic profile combines the 24 h AUC to MIC ratio (AUC_0–24_:MIC). AUC_0–24_:MIC breakpoint ≥578 mg/L.h is required in critically ill patients.^1^

**Objectives:**

Compare the model fit accuracy and breakpoint attainment using vancomycin dose and administration times recorded by infusion pumps and an ePMA system.

**Methods:**

An observational study involving critically ill adults and children treated with vancomycin extracted data from participants’ nurse-programmed infusion pumps, ePMA system documentation, and up to 15 individually taken blood samples. Optimum structural models were built using population-pharmacokinetic software (nlmixr2). Model fit assessed using objective function value (OFV). Individual AUC_0–24_:MIC calculated using isolate or empirical 1 mg/L MIC, model outputted clearance and prescribed dose(s) within initial 24 h of treatment.

**Results:**

The paediatric ePMA informed 1-compartment model (OFV=354) gave a better fit than the pump 2-compartment model (OFV=362). The adult pump 2-compartment model (OFV=242) provided a better fit than the ePMA model, whereby only a 1-compartment could be stabilized (OFV=259). Paediatric participant breakpoint attainment was 30% (3/10) using the ePMA model and 50% (5/10) using the pump model. In adults, breakpoint attainment was 33% (4/12) with the pump model compared with 50% (6/12) using the ePMA model. The pump model predicted suboptimal breakpoints in the two adults encountering vancomycin resistance, unlike the ePMA model.

**Conclusions:**

Administration data source affects model fitting and therefore breakpoint calculation. Recording errors related to infusion pump programming constraints, infusion type and setting are responsible. Pharmacokinetic models built using routine clinical data should state sources and implement data accuracy integrity measures.
